# Synthesis and Characterization of a Bioartificial Polymeric System with Potential Antibacterial Activity: Chitosan-Polyvinyl Alcohol-Ampicillin

**DOI:** 10.3390/molecules23123109

**Published:** 2018-11-28

**Authors:** Andres Bernal-Ballen, Jorge Lopez-Garcia, Martha-Andrea Merchan-Merchan, Marian Lehocky

**Affiliations:** 1 Grupo de Investigación en Ingeniería Biomédica, Vicerrectoría de Investigaciones, Universidad Manuela Beltrán, Avenida Circunvalar No. 60-00, Bogotá 1101, Colombia; 2 Centre of Polymer Systems, University Institute, Tomas Bata University in Zlín, Trida Tomase Bati 5678, 76001 Zlín, Czech Republic; lopez@utb.cz (J.L.-G.); lehocky@utb.cz (M.L.); 3 Maestría en Educación, Universidad Antonio Nariño, Conciencia, Calle 22 sur No. 12D-81, Bogotá 1101, Colombia; mmerchan30@uan.edu.co

**Keywords:** bio-artificial polymeric system, health-care associated infections, polyvinyl alcohol, chitosan, ampicillin

## Abstract

Bio-artificial polymeric systems are a new class of polymeric constituents based on blends of synthetic and natural polymers, designed with the purpose of producing new materials that exhibit enhanced properties with respect to the individual components. In this frame, a combination of polyvinyl alcohol (PVA) and chitosan, blended with a widely used antibiotic, sodium ampicillin, has been developed showing a moderate behavior in terms of antibacterial properties. Thus, aqueous solutions of PVA at 1 wt.% were mixed with acid solutions of chitosan at 1 wt.%, followed by adding ampicillin ranging from 0.3 to 1.0 wt.% related to the total amount of the polymers. The prepared bio-artificial polymeric system was characterized by FTIR, SEM, DSC, contact angle measurements, antibacterial activity against *Staphylococcus aureus* and *Escherichia coli* and antibiotic release studies. The statistical significance of the antibacterial activity was determined using a multifactorial analysis of variance with ρ < 0.05 (ANOVA). The characterization techniques did not show alterations in the ampicillin structure and the interactions with polymers were limited to intermolecular forces. Therefore, the antibiotic was efficiently released from the matrix and its antibacterial activity was preserved. The system disclosed moderate antibacterial activity against bacterial strains without adding a high antibiotic concentration. The findings of this study suggest that the system may be effective against healthcare-associated infections, a promising view in the design of novel antimicrobial biomaterials potentially suitable for tissue engineering applications.

## 1. Introduction

Copious literature has evidenced the extraordinary applicability of polymers in medical fields [1,2,3,4,5,6,7,8,9,10]. In fact, these materials are used in tissue engineering, implantation of medical devices, artificial organs, prostheses, ophthalmology, dentistry, and bone repairing [7]. Nevertheless, natural polymers do not have appropriate mechanical properties whereas synthetics are deficient in terms of biocompatibility. In this matter, their combination has gathered growing interest from the scientific community in the recent past. Most of these systems, called bioartificial polymeric systems (BAPS), have attracting characteristics for biomedical applications, and were originally conceived with the aim of combining the features of synthetic polymers (good mechanical properties, easy processability, low production and transformation costs) with the specific tissue- and cel- compatibility of biopolymers [11,12].

The BAPS may be produced as hydrogels, films, scaffolds, and a great variety of potential applications has been reported including dialysis membranes, artificial skin, cardiovascular devices, implants, bandages, or even controlled drug-release systems [13,14,15]. In spite of the remarkable potential that the BAPS have revealed, they lack inherent attributes such as bactericidal or bacteriostatic properties, whereby the BAPS in the medical field might be focused on the appeasement of healthcare-associated infections which boosts health systems costs. As an approach for facing bacteria colonization, controlled released drug systems from polymeric matrixes might be catalogued as an auspicious solution [16,17].

One of the methods for reducing or preventing infections is the development of polymer-antibiotic systems [17]. BAPS might be included in this category because of the individual properties of both natural and synthetic components, as well as the interaction that might occur among them. In that respect, chitosan (CHI) as a natural polymer has been investigated and its antimicrobial properties against a wide range of target microorganisms (algae, bacteria, yeasts and fungi) have been reported in experiments involving *in vivo* and *in vitro* studies, and in different forms (solutions, films, and composites). Nonetheless, recent data in the literature have the tendency to characterize chitosan as bacteriostatic rather than bactericidal. This polymer has been extensively used in biomedical applications, as material for chemicals encapsulation and controlled release [18,19]. CHI is widely-used as an antimicrobial agent either alone or blended with other natural polymers (starch, gelatin, alginates), in food, pharmaceutical, textile, agriculture, water treatment, and cosmetics industries [20]. Furthermore, it is hydrophilic; hence, CHI films are poor barriers to moisture. For these reasons, some biodegradable synthetic polymers, such as poly (caprolactone), poly (lactic acid), and poly (vinyl alcohol) (PVA) were used to modify strength, water resistance, and thermal stability of CHI films [21,22,23,24].

As a synthetic polymer, PVA has centered the interest from scientists in diverse areas such as engineering, chemistry and medicine. In medical uses, properties such as a high capability of water absorption, chemical resistance, physical properties, biocompatibility, and complete biodegradability are responsible for its relevant uses. PVA has indeed unique features including excellent film-forming properties and is non-toxic, as well as its significance as a part of controlled drug delivery systems, dialysis membrane, wound dressing, artificial cartilage, and tissue engineering scaffold [25,26,27].

The combination of CHI and PVA may have beneficial effects on the biological characteristics of the blend. In fact, it has been reported that the chemical crosslinking between CHI and PVA may improve the mechanical strength and thermal stability, keeping the intrinsic properties of transparency and the swelling ability of CHI films [28,29]. Indeed, the use of plasticizers and crosslinkers imparts specific properties in the system and therefore, their mechanical drawbacks are diminished or attenuated.

On the other hand, it is generally assumed that the polycationic nature of CHI, conveyed by the positively charged -NH_3_^+^ groups of glucosamine, might be a fundamental factor contributing to its interaction with negatively charged surface components of many fungi and bacteria, causing substantial cell surface alterations, leakage of intracellular substances, and ultimately resulting in impairment of vital bacterial activities [30]. Consequently, it is expected that polymers with higher charge densities resulted in an improved antimicrobial activity. Indeed, there are successful studies related to PVA/CHI systems, which show adequate cell viability, non-toxicity, and suitable properties which can be tailored for prospective uses in tissue engineering [19,29,31,32].

Despite the remarkable properties that the individual polymers evince, reinforce such properties by adding an antibiotic might be deemed as an interesting approach for treating bacteria colonization. In this matter, sodium ampicillin (AMP) is used to treating urinary infections, salmonellosis, *Listeria* meningitis, and periodontitis among others which implies that AMP has a broad target for both Gram-positive and some Gram-negative bacteria [17].

For all the aforementioned reasons, this work deals with the synthesis and characterization of a BAPS as well as the evaluation of their antibacterial activity against *Staphylococcus aureus* and *Escherichia coli*.

## 2. Results and Discussion

### 2.1. Fourier Transform Infrared (FTIR)

**Figure 1:**
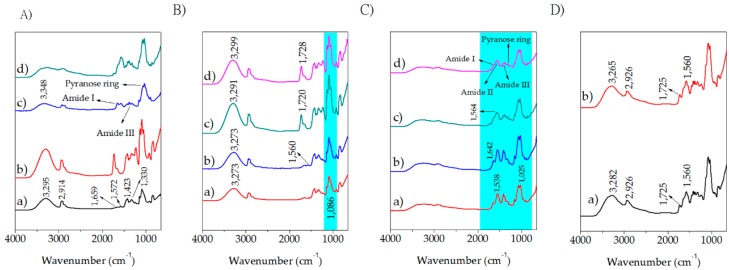
FTIR spectra for BAPS. (**A**). Polymers with and without lactic acid and glutaraldehyde (ADD): (a) PVA; (b) PVA/ADD; (c) CHI; (d) CHI/ADD. (**B**) PVA blends: (a) PVA/AMP 0.3%; (b) PVA/AMP 1%; (c) PVA/ADD/AMP 0.3%; (d) PVA/ADD/AMP 1.0%; (**C**) CHI blends: (a) CHI/AMP 0.3%; (b) CHI/AMP 1.0%; (c) CHI/ADD/AMP 0.3%; (d) CHI/ADD/AMP 1.0%; (**D**) PVA/CHI blends: (a) PVA/CHI/ADD/AMP 0.3%; (b) PVA/CHI/ADD/AMP 1.0%.

Attenuated Total Reflectance Fourier Transform Infrared (ATR-FTIR) spectra of the assessed samples are shown in Figure 1. Pristine PVA (Figure 1A(a)) displays a large band observed between 3550 and 3200 cm^−1^, which is linked to the stretching O–H from the intermolecular and intramolecular hydrogen bonds [33,34,35,36]. The band located at 2914 arises from saturated C–H stretching, whereas the band at 1423 cm^−1^ is related to –CH_2_– bending [37]. An absence of a signal in the region of 1700 cm^−1^ indicates that only a small amount of acetate groups can be present in the polymer chain as the used PVA is highly hydrolyzed. Two peaks at 1659 and 1572 cm^−1^ may be associated with conjugated diones or single carbonyls in a conjugation with C=C double bonds in a solid state, where peaks over 1700 cm^−1^ are not manifested [38]. The peak at 1155 cm^−1^ is connected to C–O stretching modes and a strong dependency of its intensity on crystallinity degree of the solid PVA material was observed [39,40]. Other authors [41,42] connect this band with C–O–C in ether bridges, and crosslinking of PVA is deduced from the increase in absorbance at this wavenumber. The band centered at 1330 cm^−1^ may surge from combination frequencies of CH and OH [43]. A strong peak located at 1060–1030 cm^−1^ is assigned for a stretching C–O in a C–O–H group [41] but the band is shifted to 1096 cm^−1^ as a consequence of interactions with unsaturated bonds [37]. The band assigned to CH_2_ rocking is located at 917 cm^−1^ whereas the signal at 854 cm^−1^ relies on C–O stretching [40].

The FTIR spectrum for CHI exhibits a broad signal centered at 3348 cm^−1^ corresponding to -OH functional groups and -NH group stretching vibrations. The symmetric and asymmetric –CH_2_– stretching occurred in the pyranose ring are located at 2920, 2880, 1430, 1320, 1275 and 1245 cm^−1^ [32,44,45]. The bands located at 1650, 1586 cm^−1^, and 1322 cm^−1^ are associated with amide I, amide II, and amide III respectively as well as the saccharine-related signals at 1155 and 900 cm^−1^ [32,46,47,48,49]. The peak at 1030 and 1080 cm^−1^ indicate the C–O stretching vibration.

The addition of lactic acid (LA) as a plasticizer, and glutaraldehyde (GLU) as a crosslinker (hereinafter as ADD) does not alter significantly the spectra (Figure 1A(a,b)). A growth of OH peaks is a consequence of the presence of LA. The C=O band at approximately 1720 cm^−1^ may denote that the aldehyde groups of GA did not completely react with the -OH groups of the PVA chain or the contribution of remaining acetate groups of the PVA structure. In addition, the C–O stretching at 1088 cm^−1^ in pure PVA is replaced by a broader absorption band located between 1000 and 1140 cm^−1^, which can be attributed to the ether (C–O) and the acetal ring (C–O–C) bands formed by the crosslinking reaction of PVA with GA. It can be assumed that GA has acted as a chemical crosslinker among PVA polymer chains [50]. By comparing CHI and CHI/ADD (Figure 1A(c,d)), a minor rise in the hydroxyl groups signal is evident. Nonetheless, the combination of both GLU and LA has contradictory effects; on one side, the crosslinker diminished the hydroxyl groups, but on the other, the plasticizer increased them. For that reason, there is no clear tendency depicted in the spectra. An increase of the signal for the imine bands as well as a drop on the amine are caused by the nucleophilic addition of the amine from CHI with the aldehyde [31].

AMP shows an absorption peak in the region of 1730–1720 cm^−1^ (Figure 1B–D), which is caused by C=O β-lactam stretching. The peaks at 1664 and 1560 cm^−1^ belong to C=O amide stretching and -NH amide groups respectively [51]. By virtue of no other modifications in the spectra, it is plausible to infer that no chemical interactions are occurring between PVA and CHI with AMP, which might imply that the antibiotic can be released from the matrix and even that the structure does not suffer significant modifications and no alterations on the antibacterial activity of AMP whatsoever. The same features are evidenced in the spectra for CHI/ADD/AMP as well as PVA/CHI/ADD/AMP.

Blends of PVA/CHI show two bands around 1640 and 1560 cm^−1^, which are connected to symmetric stretching and bending of acetamide groups, respectively. The change in the characteristic shape of the CHI spectra, as well as the shifting of peak to a lower frequency range on account of hydrogen bonding between -OH of PVA and -OH or -NH_2_ of chitosan were observed in the blended films [52]. These results suggested the formation of hydrogen bonds between the CHI and PVA molecules [53]. Special features are exhibited for the BAPS (Figure 1D). By comparing the CHI spectrum with that obtained for the BAPS, an increase of the signal for the imine peak as well as a drop on the amine signal caused by the nucleophilic addition of the amine from CHI with the aldehyde (crosslinking reaction with GLU) are presented [31]. Furthermore, a carboxylic acid dimer, which is manifested in the region of 1700–1725 cm^−1^ are originated from the acetic acid used for dissolving CHI and the absorption at 3300–3250 cm^−1^ related to -OH and -NH stretching vibrations broadened and shifted to a lower wavenumber suggests the formation of hydrogen bonds between CHI and PVA [54].

### 2.2. Scanning Electron Microscopy (SEM)

**Figure 2:**
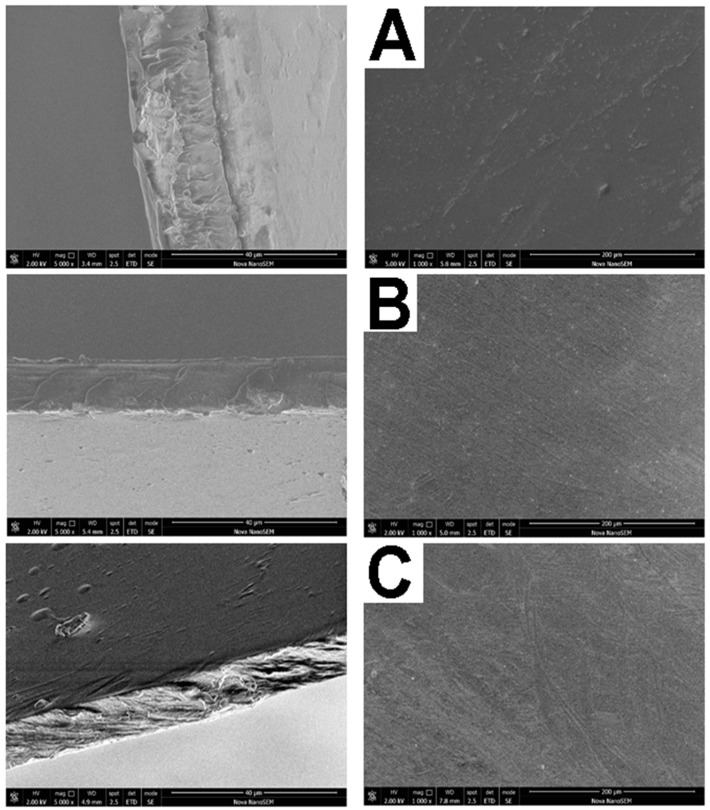
Cross section and surface SEM images of the Bioartificial polymeric systems (BAPS): (**A**) PVA/ADD/AMP. (**B**) CHI/ADD/AMP. (**C**) PVA/CHI/ADD/AMP.

Figure 2 shows the surface and cross section of scanning electron microscopy (SEM) images of PVA/ADD/AMP, CHI/ADD/AMP and PVA/CHI/ADD. No obvious agglomeration particles were observed, suggesting well-dispersed components as well as a homogeneous structure with no phase separation. The surface anomalies may be probably accounted for manipulation or storage; likewise, samples based on CHI possess wavy areas, which may be ascribed to solvent evaporation.

With regard to the cross-section images, they depict that all of the specimens have gone through a homogeneous and effective mixing process since there are no special moieties in the sample and they look considerably uniform. It is well-established that LA plasticizes the material and its presence increases the toughness. On the contrary, GLU promotes brittleness. The SEM pictures are in total correspondence with the obtained results from the FTIR spectra where there is a defined intermolecular interaction between PVA and CHI; however, this mentioned interaction is not a physical barrier, which could interfere with the release of AMP from the obtained films.

### 2.3. Differential Scanning Calorimetry (DSC)

**Figure 3:**
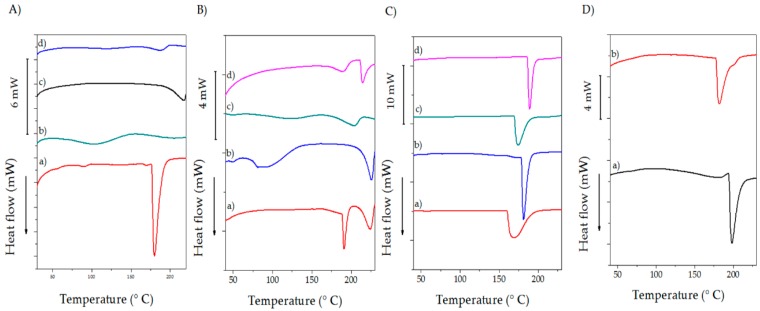
Thermogram for BAPS. (**A**). Polymers with and without ADD: (a) CHI; (b) CHI/ADD; (c) PVA; (d) PVA/ADD; (**B**) PVA blends: (a) PVA/AMP 0.3%; (b) PVA/AMP 1%; (c) PVA/ADD/AMP 0.3%; (d) PVA/ADD/AMP 1.0%; (**C**) CHI blends: (a) CHI/AMP 0.3%; (b) CHI/AMP 1.0%; (c) CHI/ADD/AMP 0.3%; (d) CHI/ADD/AMP 1.0%; (**D**) PVA/CHI blends: (a) PVA/CHI/ADD/AMP 0.3%; (b) PVA/CHI/ADD/AMP 1.0%.

The thermograms for the prepared samples are depicted in Figure 3 and they correspond to the first heating scan. The glass transition temperature (T*_g_*) (72 °C) and melting point (T*_m_*) (218 °C) for PVA (Figure 3A(a)) have been reported in several publications and the results are in a good concordance with those obtained in the performed experiment [55,56]. The presence of LA and GLU influence the thermal features of the samples. Thus, the plasticizer reduces the number of lattices, causing a decrease in the endothermic peak compared with pristine PVA, whereas the low concentration of the crosslinker in comparison to LA produces a partial grafted hydroxyl group of PVA [1]. As a result, blends of PVA/ADD melted at lower temperatures (Figure 3A(d)).

The thermograms exhibit a second endothermic peak, which is ascribed to the melting of the other minor crystalline phases. Simultaneously, no evidence of transitions related to AMP is manifested in the thermograms due to its low concentration in the samples, and because the reported T*_m_* of the antibiotic (220 °C) [57,58] is hidden by the endothermic manifestation for PVA.

CHI shows an endothermic transition between 100–120 °C that might be attributed to the evaporation of residual water [44] whereas a sharp peak at 180 °C does not correspond to T*_m_* since a recrystallization peak neither appears in the second cooling nor in the second heating scan [59]. The effects of ADD are visible in the thermograms as the amine functional groups of CHI are more reactive to GLU than the hydroxyl groups of PVA [32,60]. Therefore, a rise in the crystalline region is manifested. Furthermore, the crystallinity decreases as the crosslinking degree by GLU increases, since crosslinks between two CHI units or pendant GLU with one aldehyde free may constitute an obstacle to chitosan molecule packing. As in the case of PVA samples, the T*_m_* of AMP is not visible because of peak overlapping. BAPS exhibit endothermic peaks which started from room temperature and lasted at around 150 °C. This peak can be attributed to the presence of water molecules inside the PVA and CHI structure including tightly bound water which is released at about 100 °C, and the free loosely bound water, which exited at around 150 °C [61]. A sharp endothermic peak near to 200 °C belongs to T*_m_* of the blend since the endothermic peaks for PVA shifted towards lower temperatures by the presence of CHI [53]. The reduction of T*_m_* indicates a good miscibility of the polymers that affects the crystallization process of PVA. This drop of either the crystallization or melting temperature is considered as a measure of the blend compatibility [62].

### 2.4. Surface Wettability

**Figure 4:**
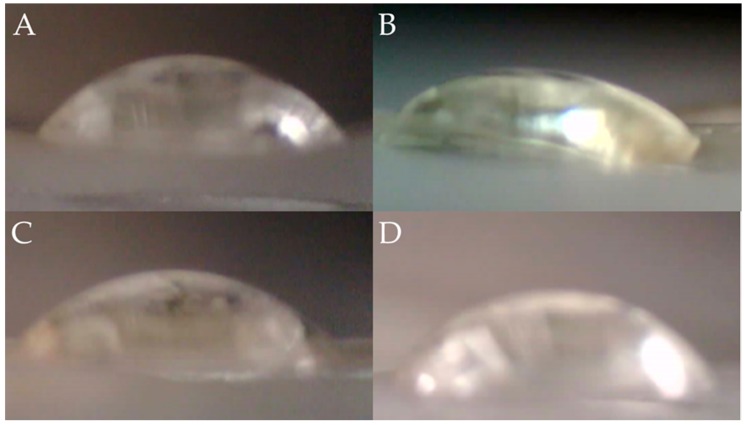
Images for contact angle measurements. (**A**) PVA/CHI/ADD/AMP 0.3% (ethylene glycol); (**B**) PVA/CHI/ADD/AMP 0.3% (diiodomethane); (**C**) PVA/CHI/ADD/AMP 1.0% (ethylene glycol) (**D**). PVA/CHI/ADD/AMP 1.0% (diiodomethane).

In order to estimate the extent of hydrophilicity, contact angle measurements were carried out. The contact angle values of deionized water (θ_w_), ethylene glycol (θ_e_), and diiodomethane (θ_d_) are reported in Table 1. Figure 4 exhibits the images taken for BAPS. Solid surfaces may be classified into two basic groups, hydrophilic (wettable with water and high surface energy) and hydrophobic (not wettable with water and low surface energy). Since a solid surface may be considered wettable if the contact angle is less than 90°, all the samples are in fact wettable. However, it is more reasonable to describe them as hydrophilic. The hydrophilicity of PVA stems from the hydrogen bonding between free -OH groups and water molecules. This feature might be reduced as the crosslinker reduces the availability of hydroxyl groups [50] as well as for the crystallinity (see DSC results).

In the case of CHI, the hydroxyl and amino groups increase the formed hydrogen bonding and, therefore, the material exhibits hydrophilic features. The plasticizer interrupts the sequence of hydrogen bonding, the intermolecular forces are affected reducing the availability of hydrophilic groups, and the material increases the contact angle. It may be observed that the samples with ADD are relatively more hydrophobic, having water contact angles above 50°. However, it seems that after some amount of AMP the hydrophilicity diminished again as a consequence of the intermolecular attractions that occur between the polymer and the antibiotic. Within this context, it is relevant to point out that a hydrophilic surface would offer a better contact with biological species and enhance the antimicrobial efficacy of the active ingredients [63]. It might explain that samples with ADD exhibit better antibacterial activity for *E. coli* than specimens that did not contain the plasticizer and crosslinker.

The presence of ADD has a higher impact on PVA than on CHI. As can be seen, a rise in the contact angle values is reported for samples containing PVA, including BAPS. It could indicate that the interactions between ADD and PVA are more effective in comparison to CHI. 

### 2.5. Microbiological Testing

**Figure 5:**
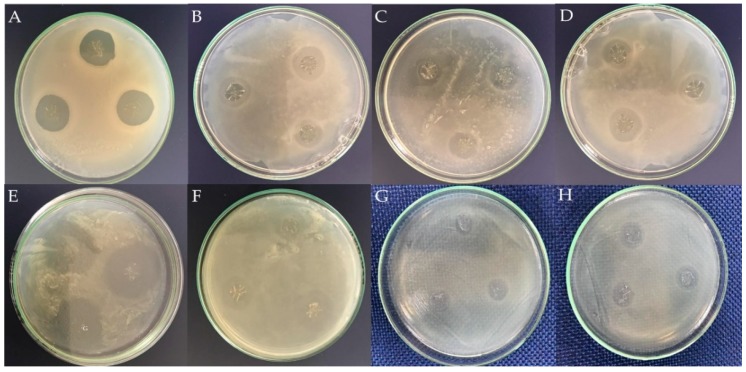
Inhibition zone for BAPS. (**A**,**B**) PVA/AMP 1.0% against *Staphylococcus aureus* and *Escherichia coli*. (**C**,**D**) PVA/ADD/AMP 1.0% against *Staphylococcus aureus* and *Escherichia coli*. (**E**,**F**) CHI/ADD/AMP 1.0% against *Staphylococcus aureus* and *Escherichia coli*. (**G**) PVA/CHI/ADD/AMP 0.3% against *Staphylococcus aureus* (**H**) PVA/CHI/ADD/AMP 1.0% against *Staphylococcus aureus*.

The method for evaluating the antibacterial activity was an agar diffusion test. The obtained results are reported in Table 2 and the inhibition halo of the tested materials are presented in Figure 5. As it can be expected, PVA did not show antibacterial activity whereas CHI requires a deeper sight. The antibacterial properties of this natural polymer depend on the molecular weight and generally show stronger bactericidal effects for Gram-positive than Gram-negative bacteria [64]. Furthermore, CHI is prepared in acetic acid, a substance which has antimicrobial activity, and bacteria in different growth stages have different sensitivity to CHI [65]. Indeed, it has been demonstrated that its antibacterial activity depends on the strain examined and on its growth phase, besides the concentration, degree of deacetylation, pH, temperature, and medium composition [66]. Then, it is plausible to affirm that the prepared samples of CHI do not have the appropriate conditions to be effective against the tested bacteria, although the presence of AMP might enhance its inherent bactericidal feature.

By comparing the presence of AMP in CHI and PVA against *Staphylococcus aureus* it is remarkable that at the same concentration of the antibiotic, the antibacterial activity of CHI is higher than PVA which might indicate that the bactericidal or bacteriostatic properties of CHI are reinforced by the presence of AMP. The combination of polymers with ADD increases the positive charges for CHI, which are responsible for its bactericidal features [67,68]. Therefore, CHI/ADD presents a higher antimicrobial activity than PVA/ADD. It may be also a consequence of synergic effects; for instance, this phenomenon is typically observed with phytochemicals, where certain molecules elicit greater influence as a group than as individual entities [67,68,69].

Three mechanisms have been proposed as an explanation for the antimicrobial properties of CHI. In the first one, the positive charges presented in the polymeric chain of CHI, on account of its amino group, interact with the negative charges from the residues of macromolecules in the membranes of microbial cells, interfering with a nutrient exchange between the exterior and interior of the cell. These charges may also compete with calcium for the electronegative sites in the membrane, compromising its integrity and causing a release of intracellular material and cellular death. The second mechanism states that CHI acts as a chelating agent, creating compounds from traces of metals essential to the cell, while the third mechanism asserts that CHI of low molecular weight is capable of entering into the nucleus of the cell, interacting with DNA, interfering with messenger RNA synthesis, affecting the synthesis of proteins, and inhibiting the action of various enzymes [18].

The molecular weight and the presence of ADD can alter the antimicrobial activity of CHI [24]. Nevertheless, the obtained results suggest that crosslinked CHI did not alter significantly their antibacterial capability. Furthermore, highly deacetylated CHI (75–85% in this research) exhibits a stronger antimicrobial activity than those with a higher proportion of acetylated amino groups, due to an increased solubility and a higher charge density [70].

It is relevant to emphasize that as far as inhibition is concerned, the highest amount of AMP exhibits the most effective antibacterial activity indicating that the antibiotic is released from the polymer matrix and it retains its antibacterial capacity. However, it is remarkably important that the prepared BAPS disclosed moderate antibacterial activity against bacterial strains without adding an extremely high concentration of antibiotic. In this matter, it has been established that AMP as a pure drug exhibits a minimum inhibitory concentration (MIC) of 3.13 and 0.20 μg/mL against *E. coli* and *S. aureus* strains [71]. For that reason, in samples with the same antibiotic concentration, the inhibition halo is higher for *S. aureus* than for *E. coli*. In the present study, the concentration of AMP was in the range of 30–100 μg/mL although it is plausible to infer that a minor amount of antibiotic is released from the matrix. That amount is located primarily on the surface of the film while the rest remains in the bulk, which can be delivered into the media once the sample dissolves as a consequence of the interaction with the solvent. In the same way, the samples that were crosslinked are diluted to a lesser extent, the antibiotic has a more complex pathway to get the exterior of the system. However, the effect of the plasticizer (an increase of the free spaces of the molecules) allows the diffusion of water and a greater amount of antibiotic can be released.

A difference in efficacy of Gram-positive and Gram-negative microorganism can be explained by the variance in the structure of the bacterial cell wall. The Gram-positive cell wall has a thicker peptidoglycan layer whereas the Gram-negative cell wall has one additional outer membrane [63]. That membrane impedes the action of the antibiotic. Although the results were consistent with the features for CHI and PVA, it is necessary to explain that AMP was effective against *E. coli* in PVA but not in CHI. *E. coli* cells are considered moderately hydrophilic whereas *S. aureus* cells exhibited more hydrophobic characteristics [72]. In this matter, the hydrophilic characteristics of PVA promote the liberation of AMP, which might interact ti a greater extent with this bacterium. On the contrary, CHI presents a lower hydrophilicity in comparison to PVA, resulting in a more complicated mechanism for AMP liberation. On the other hand, surface adherence is a natural tendency which is inherent to bacteria and other microorganisms. It has four basic steps: adhesion, colonization, formation and the subsequent bacterial biofilm growth. Biofilms act as a defense mechanism against external agents; in consequence, the aim of any antimicrobial materials is preventing bacterial adhesion and colonization, which are prerequisites to biofilm formation [73]. Therefore, any modification in the hydrophilicity of the surface will have repercussions for biofilm formation and the bacterial are more exposed to the action of the antibiotic. The use of ADD affected both PVA and CHI whether causing a reduction in the concentration of amino groups [48,74], or a lower content of hydroxyl groups [1]. Yet, interactions between the hydroxyl groups of PVA and the amino or hydroxyl groups of CHI might reduce the overall hydrophilicity of the system. Thus, the obtained results might be a consequence of the action of the released AMP, the hydrophilicity of the system, and the smoothness of the surface (see SEM images).

Two systems, PVA/CHI/ADD/AMP 0.3% and 1.0% did not reveal any inhibition over *E. coli*. As it might be foreseen, the difference between the expected and the obtained results may be ascribed to the fact that CHI hinders the adhesion of a Gram-positive strain but does not behave satisfactorily against Gram-negative bacteria, which may explain why the inhibition on PVA substrates is slightly better than on the CHI ones [75,76]. Moreover, the model of the wall of *E. coli* remains unsolved [77] although it has been established that the mechanism of AMP activity affects the cell wall synthesis as well as the acquisition of resistance of this microorganism against the mentioned antibiotic [78].

An ANOVA test shows that AMP reinforces the antibacterial activity of CHI, whereas PVA and ADD reduce slightly that property. Nonetheless, PVA and ADD enhance the poor mechanical properties of CHI and the combination of both consolidates the BAPS as a material with improved biological and physical features.

### 2.6. Antibiotic Release Studies

The mean cumulative mass of AMP released from the studied samples into distilled water as a function of elution time and the calculated constant of the Equation 1 are summarized in the Table 3.

**Figure 6:**
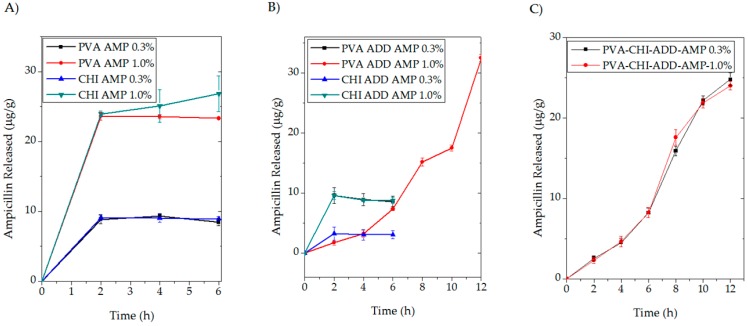
Release profile of AMP from BAPS measured by UV-VIS spectrophotometer.

According to the obtained data, the release profiles of PVA, CHI, and CHI/ADD showed a higher rate constant (k). These results might indicate that the elution of AMP from the systems occurred in the first part of the experiment. Then, as it is shown in Figure 6A,B, the released antibiotic levels off its concentration in the media, and after the third measurement no AMP was detected. These final results are consistent with the antibacterial test as well as the surface wettability which it might be related to high affinity to the polar media of the PVA, CHI and the β-lactamic antibiotic used in this study. 

The relevant results from the antibiotic release profiles focused on the BAPS were compared to all batches of samples and exhibited the smallest rate constant. In this matter of fact, the participation of ADD in the systems is related to the controlled release of AMP from the BASP which corresponded to the long-lasting antibacterial activity of the system. The results are concomitant with the antibacterial activity as well as the water contact angle results, showing the crucial role of ADD in the BAPS system likewise the synergic performance of the PVA and CHI. The stability of the samples could be also taken as outstanding results, since it showed a fast swelling due to the presence of LA, allowing the entrance of water molecules in the bulk. However, the crosslinked PVA causes a decrease in hydrophilicity, and the sustainable activity of the AMP for longer periods of time from the BASP is achieved.

## 3. Materials and Methods

Poly(vinyl alcohol) (PVA, M*_w_* 130,000 g mol^−1^) with a 99% hydrolysis, chitosan of low molecular weight and 75–85% deacetylation degree (CHI), and a 25% solution of glutaraldehyde (GLU) were provided by Sigma Aldrich, Bogotá, Colombia. Lactic acid (LA) was purchased at Fermont (Bogotá-Colombia), hydrochloric acid (HA) at Scharlau (Bogotá, Colombia), anhydrous ethylene glycol (99.8%) and diiodomethane (99%) were purchased from Sigma Aldrich (Saint Louis, MO, USA), and sodium ampicillin (AMP) was produced by Farmalógica, S.A. (Bogotá-Colombia) and donated to this research by The *Hospital Cardiovascular del Niño de Cundinamarca* (Soacha, Colombia). All of the reagents were used as they were received.

### 3.1. Sample Preparation

An aqueous solution of PVA at 1 wt.% was prepared by dissolving the polymer in distilled water for 6 h at 80 °C under continuous magnetic stirring (ThermoScientific—SP131325Q, Shangai, China). Thereupon, LA at 5 wt.%, HA at 1.2 wt.%, and GLU at 0.25% with respect to the total amount of the polymer were added as plasticizer and crosslinker agents and the solution was stirred for 20 more min. CHI was dissolved in acetic acid (0.5 M) at room temperature by the slow addition of the polymer to the solvent under mild magnetic stirring (ThermoScientific—SP131325Q, Shangai, China) for getting a 1 wt.% solution. Then, GLU (0.25 wt.%), HA (1.2 wt.%), and LA (5 wt.%) related to the total amount of the polymer were added as crosslinker and plasticizer agents. Blends of a 1:1 ratio of PVA and CHI were prepared and stirred until a homogeneous appearance with and without ADD followed by the addition of AMP (0 to 1 wt.%, related to the polymer). The solutions were stirred for 10 min and films were obtained using the casting method pouring 0.5 mL per cm^2^ on plastic Petri dishes and the films were allowed to dry at 37 °C for a week in a no-air circulating oven.

### 3.2. Fourier Transform Infrared (FTIR)

FITR spectroscopy analysis was carried out on NICOLET 6700 FTIR spectrometer device (Thermo scientific, Waltham, MA, USA) equipped with attenuated total reflectance (ATR) accessory utilizing the Zn–Se crystal and software package OMNIC over the range of wavelengths from 4000 to 600 cm^−1^ at room temperature under a resolution of 4 cm^−1^. Each spectrum represents 64 co-added scans referenced against an empty ATR cell spectrum.

### 3.3. Scanning Electron Microscopy (SEM)

Micrographs of the prepared samples were taken by the scanning electron microscope Nova NanoSEM 450 (FEI, Brno, Czech Republic) with a Schottky field emission electron source operated at an acceleration voltage ranging from 200 V to 30 kV and a low-vacuum SED (LVD) detector. A coating with a thin layer of gold was performed by a sputter coater SC 7640 (Quorum Technologies, Newhaven, East Sussex, UK).

### 3.4. Differential Scanning Calorimetry (DSC)

Calorimetric measurements were carried out in a DSC 1 calorimeter, Mettler Toledo (Greifensee, Zurich, Switzerland), under nitrogen flowing at a rate of 30 mL min^−1^. The specimens were pressed in sealed aluminum pans. A heating cycle was performed in order to acquire the glass transition temperature (T*_g_*) and melting temperature (T*_m_*). The samples were cooled down by nitrogen at an exponentially decreasing rate. The heating of the cycle was performed from 25 to 250 °C at a rate of 20 °C/min. The T*_g_* was determined as the midpoint temperature by standard extrapolation of the linear part of DSC curves using Mettler-Toledo Stare software and the T*_m_* as the maximum value of the melting peak.

### 3.5. Surface Wettability

The wettability of the samples was evaluated by contact angle measurement. The sessile drop method was employed for this purpose on a Surface Energy Evaluation (SEE) system equipped with a CCD camera (Advex Instruments, Brno, Czech Republic). Deionized water, diiodomethane, and anhydrous ethylene glycol were used as testing liquids at 22 °C and 60% relative humidity. The droplets volume was set to 5 μL for all experiments. Every representative contact angle value was an average of 5 independent measurements.

### 3.6. Microbiological Tests

The antibacterial properties of the films were assessed by using the agar diffusion test. Round specimens (8 mm in diameter) were placed on the surface of an individual nutrient agar plate, where a bacterial solution of chosen microorganisms had been swabbed uniformly (*Staphylococcus aureus*-ATCC 9144, and *Escherichia coli*- ATCC 11775). After 24 h incubation at 37 °C, the dimensions of the inhibition zones were measured in four directions, and the average values were used to calculate the circle zone inhibition area.

### 3.7. Antibiotic Release Studies

To measure the AMP release, round shaped samples (15 mm in diameter) were immersed into 10 mL distilled water at 37 °C with continuous shaking (100 rpm). After defined periods of time, the samples were transferred into the fresh medium to reach perfect sink conditions. The released AMP in the elutions was detected by UV-vis spectrophotometer (Thermo Scientific, Helios Gamma, Waltham, MA, USA) at a wavelength of 210 nm. Calibration dependences of the absorbance (A) on AMP concentration (C_AMP_ in μg·L^−1^) for release in distilled water (A = 0.0528 C_AMP_ + 0.0436, R^2^ = 0.9984) were determined prior to the release investigation. Afterwards, the cumulative mass was calculated. The measurements were performed by triplicate. The observed data of the cumulative mass of the released AMP related to 1 g of the sample material were evaluated by using first-order kinetics (Equation (1)) and regression processed by the least squared where C_REL_ (μg/g) is the experimental concentration of AMP that was released at time t, C_MAX_ (μg/g), means the maximal theoretical concentration of the AMP released from 1 g of the sample, and −k(h^−1^) represents the rate constant i.e., time needed to reach C_MAX_.

C_REL_ = C_MAX_ × (1 − e^−kt^) (1)

### 3.8. Statistical Analysis

Microbiological tests were performed in quintuplicate and the experimental values are reported in form of average ± standard deviation. Results were statistically compared applying a multifactorial analysis of variance (ANOVA) with *p* < 0.05 with SPSS software.

## 4. Conclusions

This contribution delved into the incorporation of sodium ampicillin to PVA/CHI system. The studied-referred to herein as BAPS were uniform and stable with interesting antibacterial results. It was observed throughout this study that the presence of AMP is different in each system, where under the same concentration of the antibiotic, the antibacterial activity of CHI was higher than PVA which might indicate that the bactericidal or bacteriostatic properties of CHI were reinforced by synergic effects with AMP. Indeed, it is plausible to infer that AMP might be released from the polymer matrix and the antibiotic keeps its antibacterial activity. The prepared BAPS endorse an interesting breakthrough in the development of novel antimicrobial biomaterials potentially suitable for tissue engineering applications, since the samples with only 0.3% of AMP showed effectiveness against both Gram-positive and Gram-negative bacterial strains without adding a high antibiotic concentration.

Apart from the microbiological test, succinct spectroscopic, topographical and thermal assessments have been made shedding light on the chemical and thermal variations which occur when any of the starting material is added. Polymer science and material innovation is undeniably a path towards creativity with eminent progress in the last years and with great challenges to overcome and the bio-artificial polymeric systems appraise here are indeed a promising approach for tissue engineering applications. Hence, the findings of this study open the door to more in-depth research both from scientific and medical standpoints.

## Figures and Tables

**Table 1:** Contact angle values for the prepared materials.

Description	Contact Angle (°)		
	*Water*	*Ethylene glycol*	*Diiodomethane*
PVA	<10 *	<10	34.93 ± 5.33
CHI	<10 *	<10	45.33 ± 1.71
PVA/AMP 0.3%	<10 *	***	35.24 ± 4.97
PVA/AMP 1.0%	<10 *	***	41.46 ± 3.82
CHI/AMP 0.3%	<10 *	55.76 ± 4.75	45.55 ± 3.34
CHI/AMP 1.0%	<10 *	47.73 ± 6.54	45.56 ± 3.02
PVA/ADD/AMP 0.3%	61.14 ± 4.13 **	***	22.94 ± 2.00
PVA/ADD/AMP 1.0%	43.12 ± 1.62 **	***	34.35 ± 2.59
CHI/ADD/AMP 0.3%	<10 *	***	45.51 ± 3.45
CHI/ADD/AMP 1.0%	<10 *	***	44.24 ± 5.69
PVA/CHI/ADD/AMP 0.3%	62.43 ± 3.82 **	44.36 ± 2.65	45.06 ± 2.61
PVA/CHI/ADD/AMP 1.0%	53.91 ± 2.67 **	43.22 ± 1.93	51.58 ± 3.88

* In these cases the samples are hydrophilic and the surface got wet very quickly. ** Although the samples have a water contact angle, the specimens got swollen in approximately 5 s. *** It reacted with the solvent.

**Table 2:** Agar diffusion test of the studied sample against *Staphylococcus aureus* and *Escherichia coli*.

Sample	Inhibition Zone (mm) *Staphylococcus aureus*	Inhibition Zone (mm) *Escherichia coli*
PVA	-	-
CHI	-	-
PVA/AMP 0.3%	2.34 ± 0.69	-
PVA/AMP 1.0%	3.54 ± 0.60	1.26 ± 0.05
* CHI/AMP 0.3%	3.26 ± 0.87	-
* CHI/AMP 1.0%	4.36 ± 0.35	-
PVA/ADD/AMP 0.3%	-	1.27 ± 0.17
PVA/ADD/AMP 1.0%	1.30 ± 0.17	1.13 ± 0.05
* CHI/ADD/AMP 0.3%	3.61 ± 0.31	1.16 ± 0.21
* CHI/ADD/AMP 1.0%	4.18 ± 0.21	1.10 ± 0.16
* PVA/CHI/ADD/AMP 0.3%	1.18 ± 0.04	-
* PVA/CHI/ADD/AMP 1.0%	1.20 ± 0.10	-

* ANOVA test showed that CHI was the unique component which its significance was higher than ρ < 0.05.

**Table 3:** Release studies of AMP from the prepared samples.

% AMP	C_MAX_ (μg/g)	−k × 10^−3^ (1/h)	R^2^
PVA/AMP 0.3%	26.69 ± 0.94	43.1	0.90
PVA/AMP 1.0%	70.54 ± 0.71	53.8	0.90
CHI/AMP 0.3%	27.11 ± 0.85	45.5	0.90
CHI/AMP 1.0 %	75.90 ± 2.09	70.5	0.90
PVA/ADD/AMP 0.3%	27.13 ± 0.40	0.40	0.90
PVA/ADD/AMP 1.0%	75.60 ± 1.63 *	0.45	0.90
CHI/ADD/AMP 0.3%	27.12 ± 0.44	57.9	0.90
CHI/ADD/AMP 1.0%	73.00 ± 2.16	68.7	0.95
PVA/CHI/ADD/AMP 0.3%	78.28 ± 0.23 *	0.33	0.95
PVA/CHI/ADD/AMP 1.0%	78.74 ± 2.58 *	0.41	0.95

* These values were obtained after 24 h. The others after 6 h.
